# The Role of Exosomes and Their Cargos in the Mechanism, Diagnosis, and Treatment of Atrial Fibrillation

**DOI:** 10.3389/fcvm.2021.712828

**Published:** 2021-07-28

**Authors:** Shengyuan Huang, Yating Deng, Jiaqi Xu, Jiachen Liu, Liming Liu, Chengming Fan

**Affiliations:** ^1^Department of Cardiovascular Surgery, The Second Xiangya Hospital, Central South University, Changsha, China; ^2^Xiangya Medical College of Central South University, Changsha, China; ^3^Department of Spine Surgery and Orthopaedics, Xiangya Hospital, Central South University, Changsha, China

**Keywords:** non-coding RNA, exosomes, atrial fibrillation, biomarkers, mechanism, treatment

## Abstract

Atrial fibrillation (AF) is the most common persistent arrhythmia, but the mechanism of AF has not been fully elucidated, and existing approaches to diagnosis and treatment face limitations. Recently, exosomes have attracted considerable interest in AF research due to their high stability, specificity and cell-targeting ability. The aim of this review is to summarize recent literature, analyze the advantages and limitations of exosomes, and to provide new ideas for their use in understanding the mechanism and improving the diagnosis and treatment of AF.

## Introduction

Atrial fibrillation (AF) is the most common persistent arrhythmia. In the United States, 2.3 million people have AF, and this number is expected to increase to 5.6 million by 2050 (1). AF greatly increases the risk of stroke, with a more than five-fold excess of stroke in subjects with AF (2), and contemporary studies have shown that 20–30% of patients with ischemic stroke had been diagnosed with AF (3). AF is also one of the main causes of heart failure, sudden death, and other cardiovascular diseases (3). After adjusting for preexisting cardiovascular conditions, AF increases all-cause mortality by a factor of two (4).

AF is widespread and harmful, but current diagnostic methods are not very efficient. Twelve lead electrocardiography is currently the gold standard for diagnosis, but due to its short recording time window, paroxysmal atrial fibrillation goes undetected in many patients (5). To address this limitation, ambulatory electrocardiographic recording devices were developed (6), but they are uncomfortable for patients during recording, and even the extended recording time may be insufficient (5). Therefore, it would be helpful to develop alternative convenient and efficient diagnostic tools.

Currently, scholars believe that AF occurs when atrial tissue undergoes structural and/or electrophysiological remodeling, which can promote formation and propagation of abnormal electrical pulses (7). Atrial abnormalities can occur *via* several mechanisms, such as renin angiotensin aldosterone system (RAAS) activation, cardiac fibrosis, decreased action duration potential, and others (8). The variety of mechanisms and pathways involved in atrial fibrillation contribute to its mechanism not yet being fully understood.

As the understanding of AF has improved, many treatments have emerged, including antiarrhythmic drugs (AADs), biologicals, catheter ablation, and the COX-maze procedure (9). However, most currently available treatments have major limitations. For example, AADs can cause malignant ventricular arrhythmia and lung and hepatic damage. Catheter ablation requires a long operation, while the result is not ideal (rhythm control is achieved in only 57–80% of cases), and long-term complications often result (9, 10). Although the COX-maze procedure yields a higher rate of postoperative freedom from AF (90% after 1 year), it is mainly suitable for symptomatic patients undergoing other cardiac surgical procedures (11). Therefore, further research on treatments for atrial fibrillation is warranted.

As mentioned above, AF is associated with a high risk of stroke, and the mechanism, diagnosis and treatment of AF require further study. Currently, exosomes and non-coding RNAs (ncRNAs) are areas of active cardiovascular research. What new perspectives and ideas will exosomes and ncRNAs bring to AF? We review recent research on ncRNAs and exosomes in AF, and provide suggestions for further research and clinical applications.

## A Brief Introduction of Exosomes and Their Cargos

### Exosomes

Exosomes are extracellular vesicles that originate from endosomes and have diameters ranging from 40 to 160 nm. At the time of their discovery, exosomes were regarded as a kind of “cell junk.” Researchers believed that the role of exosomes was mainly to remove excess components in cells to maintain cell homeostasis (12). It later became clear that they have a role in intercellular communication and disease progression (13). Researchers believe that exosomes might constitute an ideal treatment because they can target specific cells and regulate complex pathways. In addition, exosomes have been detected in various body fluids (14), indicating that they have the potential to become biomarkers of multiple diseases.

**Figure 1:**
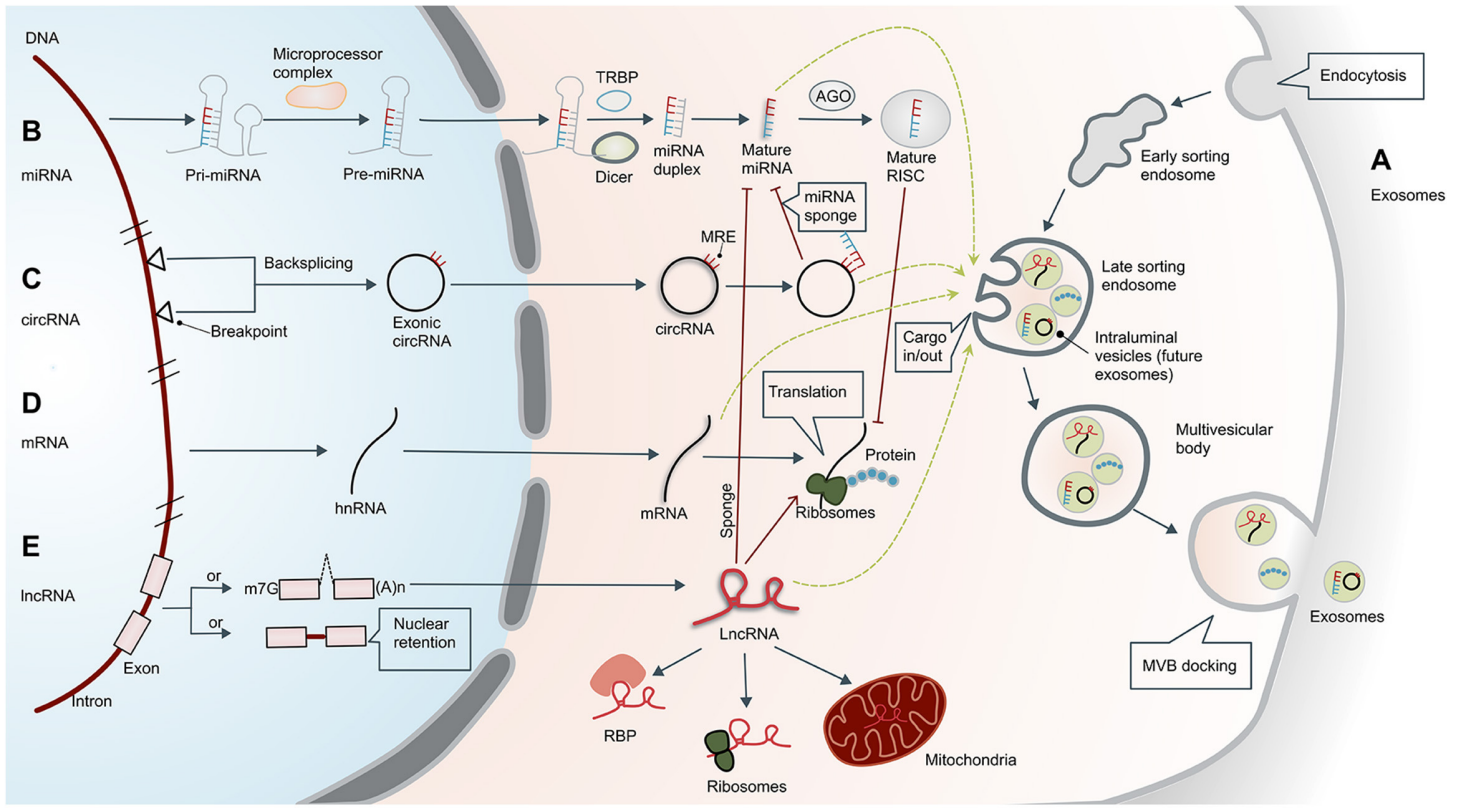
Biogenesis of exosomes and non-coding RNAs. **(A)** Biogenesis of exosomes. Formation of exosomes begins with invagination of the plasma membrane. This process produces early sorting endosomes (ESEs) with cargos of what had been extracellular material. After cargos are transferred to and from other membranous structures, ESEs become late sorting endosomes (LSEs), whose secondary invagination forms intraluminal vesicles (ILVs—the future exosomes) that give rise to multivesicular bodies (MVBs). Finally, MVBs are transferred to and dock on the plasma membrane, resulting in release of the exosomes. The exosomes usually contain mRNA, proteins, and non-coding RNAs (ncRNAs), including microRNAs (miRNAs), circular RNAs (circRNAs), and long non-coding RNAs (lncRNAs). **(B)** Biogenesis and functional mechanism of microRNAs. Primary miRNAs (pri-miRNAs) are transcribed and cleaved by the microprocessor complex to form precursor miRNAs (pre-miRNAs). Pre-miRNAs are then transported into the cytoplasm and further processed by DICER or co-factors, including the trans-activation response RNA-binding protein (TRBP). Mature miRNAs in the cytoplasm are transported to the RNA-induced silencing complex (RISC), which can bind to the Argonaute (AGO) protein and ultimately guide the complex to the target mRNA. **(C)** Biogenesis of circular RNAs. Exonic circRNAs are formed by backsplicing the transcription products between two breakpoints on DNA. CircRNAs have a closed ring structure without a 5′-end cap and a 3′-end polyadenosine tail structure. In the cytoplasm, circRNAs can act as ceRNAs by sponging miRNAs with miRNA response elements (MREs), thereby reducing the inhibitory effect of the miRNAs. **(D)** Biogenesis of mRNAs. Translation of mRNA is the key regulatory target of ncRNA, which is separate from the regulatory effect of lncRNAs and miRNAs. The mRNAs can also enter exosomes. **(E)** Biogenesis of long non-coding RNAs. Unlike mRNA, many lncRNAs are processed incompletely and remain in the nucleus, while others are spliced efficiently and transported to the cytoplasm. In the cytoplasm, lncRNAs can interact with different types of RNA-binding proteins (RBPs) and ribosomes. In addition, lncRNAs are usually sorted into mitochondria or other organelles, including exosomes. They perform post-transcriptional functions by promoting/suppressing mRNA stability or sponging miRNA as competing endogenous RNAs (ceRNAs). m7G, 7-methyl guanosine 5′ cap; (A)n, poly(A) 3′ tail.

The formation of exosomes begins with invagination of the plasma membrane. This process produces early sorting endosomes (ESEs). After cargos are transferred to and from other membranous structures, ESEs become late sorting endosomes (LSEs), whose secondary invagination forms intraluminal vesicles (ILVs). After processing and modification, ILVs become exosomes that carry a variety of cargos, including proteins, amino acids, lipids, DNA, RNA, and metabolites (15). LSEs transform into multivesicular bodies (MVBs) that dock on the plasma membrane and release the enclosed exosomes to the extracellular space (Figure 1A).

Research on cargos has concentrated on RNA, and here we focus on ncRNAs.

### Non-coding RNAs in Exosomes

Non-coding RNAs (ncRNAs) are RNA molecules that are not translated, but rather participate in post-transcriptional regulation of microRNAs (miRNAs), piwi-interacting RNAs, long non-coding RNAs (lncRNAs), circular RNAs (circRNAs), and endogenous small interfering RNAs, among others. Due to their extensive and complex regulatory effects, ncRNAs are involved in a variety of diseases, and have gradually become key molecules for diagnosis and treatment (16).

#### MicroRNAs

MicroRNAs are members of a family of small ncRNAs with a length of 18–23 bases, which are highly conserved among species (17). Although miRNAs do not code for proteins, they play important roles in inhibiting or promoting mRNA degradation to regulate gene expression (18). The occurrence and maturation of miRNAs are regulated by a variety of cellular factors, and their biosynthesis involves transcription and cleavage of primary miRNAs (pri-miRNAs) by the microprocessor complex to form precursor miRNAs (pre-miRNAs) (19). Pre-miRNAs are then transported into the cytoplasm and further processed by DICER or co-factors, including the trans-activation response RNA-binding protein (TRBP) or the protein activator of protein kinase R (PACT) (20). Mature miRNAs in the cytoplasm are then transported to the RNA-induced silencing complex (RISC), which can bind to the Argonaute (AGO) protein and ultimately guide the complex to the target mRNA (21) (Figure 1B). According to the principle of base complementarity, miRNAs directly regulate the stability of mRNA to affect cell behavior.

#### Circular RNAs

Circular RNAs are a unique class of ncRNAs. In contrast to linear RNAs, circRNAs have a closed ring structure without a 5′-end cap and 3′-end polyadenosine tail structure (22). This structure promotes cyclization of exons and/or introns to each other, potentially protecting them from degradation and providing a half-life of about 48 h (23). The circRNA sequences are highly conserved and have distinctive histological and temporal characteristics (24), participating in the regulation of transcriptional and post-transcriptional gene expression (25). CircRNAs can act as miRNA sponges, thereby reducing the inhibitory effect of miRNA and increasing expression of specific target genes (26) (Figure 1C).

#### Long Non-coding RNAs

Long non-coding RNAs are a kind of ncRNA with a length of more than 200 base pairs. Unlike mRNA (Figure 1D), many lncRNAs are processed incompletely and retained in the nucleus (27), while others are spliced efficiently and transported to the cytoplasm. In the cytoplasm, lncRNAs can interact with different types of RBPs and ribosomes. In addition, lncRNAs are usually sorted into mitochondria or other organelles, including exosomes (Figure 1E). The lncRNAs function primarily in three ways. First, they can bind transcription factors and the promoter region of target genes, which upregulates their transcription. Second, they can act like miRNA to downregulate mRNA. Third, they can target other post-transcriptional regulators such as miRNAs. The lncRNAs can act as miRNA sponges to affect mRNA levels and thus regulate gene expression (28). A more detailed description can be found in the article by Statello et al. (27). In addition to transcriptional or post-transcriptional regulation, lncRNAs also have roles in epigenetics, cellular stability, etc.

## Clinical Potential of Exosomes and Their Cargos

### The Potential of Exosomes

Researchers have extensively studied the role of exosomes in cardiovascular disease. Zeng et al. revealed that increased circulating levels of the exosomal lncRNAs ENST00000556899.1 and ENST00000575985.1 can be a potential biomarker for acute myocardial infarction (29). The study by Zhu et al. (30) showed that exosomes can also release the lncRNA MALAT1, which prevents aging-induced cardiac dysfunction by inhibiting the NF-κB/TNF-α signaling pathway. Zhou et al. (31) reviewed the role of circulating exosomes, and nine types of exosomal MiRNAs were suggested to be useful for the diagnosis and prognosis of heart failure.

### The Potential of MicroRNAs

Currently, only a small number of miRNAs have been identified. Analysis of miRNA sites in the genome showed that miRNAs play an important role in several physiological processes, including blood cell differentiation, homeobox gene regulation, neuronal polarity, insulin secretion, brain morphogenesis, and cardiogenesis (32, 33). Recent studies have identified ~50 miRNAs associated with essential hypertension, and more than 30 miRNAs associated with heart failure and myocardial infarction, many of which might serve as useful biomarkers (34–36). Some miRNAs, such as miR-29b, miR-323-5p, miR-455, and miR466, regulate expression of matrix metalloproteinase-9 in diabetic heart tissue and promote proliferation of endothelial cells (ECs) and formation of capillary-like structures, which could protect oxygen-damaged cardiomyocytes (37). Ong et al. (38) found that miR-27b-3p could target Wnt3A to regulate Wnt/β-catenin signaling in experiments using a rat model, thereby attenuating atrial fibrosis. These studies suggest that miRNA may play an important role in AF.

### The Potential of Circular RNAs

Recent studies have indicated that circRNAs are widely involved in cardiovascular disease. Huang et al. (39) showed that the absence of the Super-Enhancer-Regulated circRNA Nfix in a mouse model of myocardial ischemia promotes regeneration and repair of myocardial cells. Li et al. (40) showed that circRNA_000203 enhances Gata4 gene expression by down-regulating miR-26b-5p and miR-140-3p, thereby exacerbating cardiac hypertrophy. Other studies have also shown that circRNAs also play an important regulatory role in myocardial fibrosis and vascular regeneration (41, 42).

### The Potential of Long Non-coding RNAs

Long non-coding RNAs are widely involved in cardiovascular disease. Studies have shown that inhibition of AK088388 can increase the level of miR-30a and reduce the levels of mRNA and protein of the autophagy markers Beclin-1 and LC3-II, thus significantly reducing autophagy and cardiomyocyte damage (43). Fan et al. used microarray analysis to evaluate the variability of lncRNA in mouse aortic endothelial cells carrying vulnerable plaques, and found that the expression pattern of lncRNA UC.98 was closely related to the vulnerability of atherosclerotic plaques (44). Tao et al. found that expression of miR-21 can be down-regulated when expression of growth specific blocking factor 5 (GAS5) lncRNA is elevated, and miR-21, which promotes the progression of cardiac fibrosis, is overexpressed in cardiac fibrotic tissue and activated cardiac fibroblasts (45).

### The Advantages of Exosomes and Their Cargos for Clinical Use

The use ncRNAs, such as miRNAs, in clinical practice may encounter several problems. First, in the search for biomarkers for diagnosing AF, there have been inconsistencies between different studies (Table 1). An explanation for these inconsistencies may be that the RNAs in the circulating blood come from different tissues or cells, and other components in the blood may also affect the results. In addition, expression of miRNA is related to the heterogeneity of the population of patients with AF (50). Second, as a treatment method, miRNAs also have problems with delivery efficiency and off-target effects. Because they can target a variety of organs and tissues, miRNA mimics used for treatment may not be accurately delivered to the intended tissues (51). Thus, delivery efficiency is relatively low and may cause side effects.

**Table 1:** miRNAs as biomarkers for the diagnosis of AF patients.

**miRNAs**	**Tissue**	**Changes in AF**	**Characteristics**	**References**
miRNA-150	Plasma	Reduced	OR 1.96, 95% CI 1.5–3.57, P < 0.001	Liu et al. (46)
miRNA-29b	Plasma/Atrial	Reduced	Plasma concentrations were decreased by 53.8% in patients with AF (P = 0.007); tissue-expression was decreased by 54%	Dawson et al. (47)
miR-328	Plasma	Increased	OR 1.21, 59%CI 1.09–1.33, P < 0.001	McManus et al. (48)
miR-99a-5p,−192-5p,−214-3p, and−342-5p	Plasma	Increased	The area under the curves (AUCs) were 0.67, 0.73, 0.78, and 0.79, and the Youden's indices were 0.38, 0.44, 0.50, and 0.50, respectively.	Natsume et al. (49)

**Figure 2:**
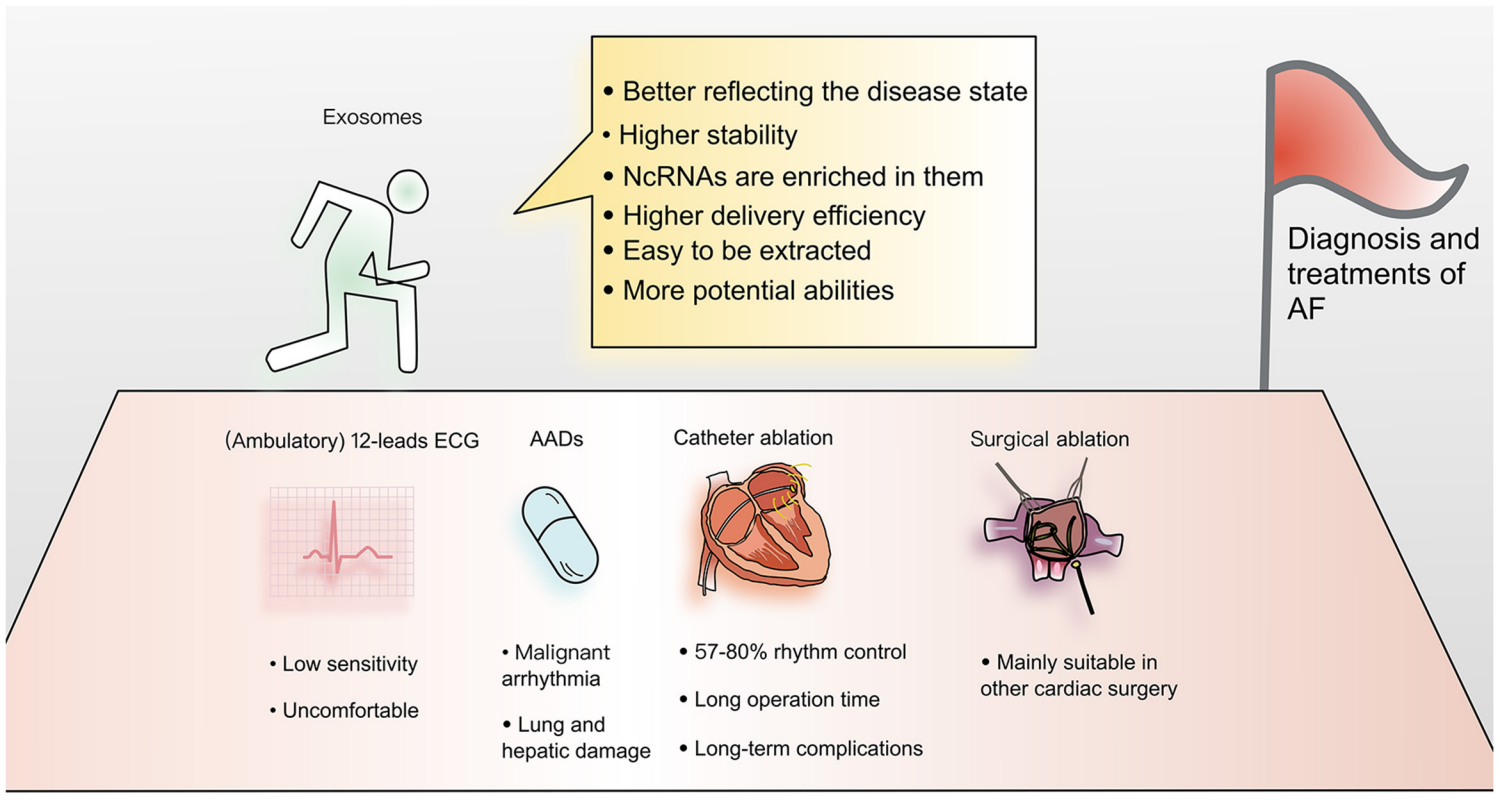
Advantages of applying exosomes to the diagnosis and treatment of atrial fibrillation. Current diagnosis and treatment methods for AF have many limitations. Exosomes have become a focus of cardiovascular research. Due to their many advantages, exosomes are thought to have the potential to overcome current limitations and provide new methods for understanding the mechanism and improving the diagnosis and treatment of AF.

However, loading ncRNA into exosomes provides several advantages for diagnosis and treatment:

1. Abnormal cells and diseased tissues tend to produce more exosomes because of changes in cytoplasmic physiology (52). So exosomes are often from abnormal tissues, better reflecting the disease state.
2. Compared with ncRNAs in biological fluids, the contents of exosomes are more stable and therefore more useful as clinical biomarkers.
3. The ncRNAs in exosomes are naturally enriched, facilitating detection.
4. Exosomes are a natural mechanism of cellular communication and molecular transportation, and deliver materials with high efficiency. This feature makes them very attractive for pharmaceutical applications.
5. Their biophysical properties make it easy to extract exosomes and manipulate their protein and RNA cargoes (53).
6. Exosomes are composed of multiple components, which means that they have greater potential to perform complex functions, such as modifying protein or lipid components to reduce adverse reactions (15) (Figure 2).

All these characteristics make exosomes more effective than free ncRNAs in the diagnosis and treatment of AF.

## Diagnostic and Therapeutic Role of Exosomes in Atrial Fibrillation

### Exosomes in the Diagnosis of Atrial Fibrillation

Since 2019, there have been attempts to identify links between exosomes and AF that might improve its diagnosis and prognosis, and most studies have focused on miRNAs in exosomes. Wang et al. compared differences in circulating exosomes between AF patients and control groups, and analyzed three differentially expressed miRNA components, miR-483-5p, miR-142-5p, miR-223-3p, and miR-223-5p. Among them, miR-483-5p was independently related to AF, revealing that exosomes have great potential for diagnosing AF (54). The studies of Wei et al. and Liu et al. are similar. The former study showed that expression of miR-92b-3p, miR-1306-5p and miR-let-7b-3p are different in patients with AF compared to those with normal sinus rhythm (55), while the latter identified miR-382-3p as a differentially expressed miRNA (56). The study of Mun et al. attempted to identify specific biomarkers for persistent AF. They identified four kinds of miRNA in exosomes (miRNA-103a,−107,−320d,−486, and let-7b) found in patients with persistent AF but not in those with paroxysmal AF or in control patients (50).

There have also been studies trying to use other components in exosomes for diagnosis of AF, such as mitochondrial DNA (mtDNA) and protein. Soltész et al. searched for differentially expressed mtDNA in exosomes of AF patients. However, in addition to finding that mtDNA levels were different in exosomes, peripheral blood and cell-free plasma, there was no obvious difference between AF patients and healthy controls, indicating that mtDNA is not suitable for use as a biomarker for AF (57). Ni et al. believed that the protein in exosomes had not been fully studied, so they analyzed the difference in protein content in exosomes between AF patients and controls. Their results showed that the difference between the proteins in exosomes of AF patients and the control group was mainly reflected in protein folding, which caused the protein content of exosomes from AF patients to be less due to protein misfolding or unfolding (58).

### Exosomes in the Pathogenic Mechanism of Atrial Fibrillation

Many studies have clarified the mechanism by which exosomes are involved in the development of AF. Recently, Shaihov-Teper et al. discovered that epicardial fat (eFat) could facilitate AF, and extracellular vesicles were one of the important mechanisms. They extracted and cultured eFat from patients with AF, then collected extracellular vesicles in the culture medium, and found that these vesicles had proinflammatory, profibrotic, and proarrhythmic effects, which ultimately promoted the formation of AF (59). Similarly, the study by Li et al. tried to reveal the mechanism by which myofibroblasts (MFBs) promote the progression of AF. They found that exosomes secreted by MFBs can act on cardiomyocytes (CMs) and cause the latter to down-regulate the expression of the L-type calcium channel Cav1.2, which is a typical AF-associated ionic remodeling marker. Further studies have shown the miR-21-3p in exosomes may be the inhibitory molecule responsible for this down-regulation (60). In addition, it has been observed that the lncRNA NRON (ncRNA repressor of the nuclear factor of activated T cells) can promote polarization of M2 macrophages to reduce atrial fibrosis, and a study by Li et al. showed that exosomes are involved in this process. The exosomes secreted by CMs stimulated by NRON can promote polarization of M2 macrophages and reduce expression of fibrosis markers in cardiac fibroblasts (61).

### Exosomes in the Treatment of Atrial Fibrillation

Although there has been no research on the application of exosomes to the treatment of AF patients, there have been studies that demonstrated a role for exosomes in the treatment of AF in mice. The miR-320d in cardiomyocytes with AF is down-regulated, which leads to increased apoptosis and decreased cell viability. Liu et al. tried to use exosomes from adipose tissue-derived mesenchymal stem cells to deliver miR-320d mimics to cardiomyocytes, and showed that the above process was reversed. These results support exosomes as an effective drug delivery system (62).

## Non-coding RNAs in Atrial Fibrillation

To the best of our knowledge, there are currently only 10 articles that directly relate exosomes with AF (discussed in the previous section). Although many ncRNAs have been successfully used to better study the mechanism and aid in the diagnosis and treatment of AF, they were not reported in exosomes in those studies. Therefore, in order to guide future research, we summarize the ncRNAs that have been studied in the context of AF but not yet reported in exosomes.

### MicroRNAs

#### MicroRNA in the Diagnosis of Atrial Fibrillation

The following miRNAs have been shown to appear in exosomes and contribute to the diagnosis of AF: miR-483-5p, miR-142-5p, miR-223-3p, miR-223-5p, miR-92b-3p, miR-1306-5p and miR-let-7b-3p, miR-382-3p, miRNA-103a,−107,−320d,−486, and let-7b (50, 54–56).

In addition, many circulating miRNAs have been shown to be associated with AF, which may guide future exosome research. McManus et al. showed miR-21 and miR-150 in the plasma of AF patients were lower than those in patients with sinus rhythm. And interestingly, the level was lower in the plasma of patients with paroxysmal AF than in those with persistent AF, which means they can not only be a biomarker for AF, but potentially distinguish AF subtypes (63).

As biomarkers, miRNAs were also found to have the potential to evaluate AF in more complex situations. Zhou et al. (64) showed that miR-21 is related to the prognosis of patients with AF after radio-frequency ablation, helping to guide physicians in choosing whether to perform that procedure. And considering miR-29s along with age and amino-terminal procollagen III peptide (PIIINP) helps to predict whether the patient will develop AF after receiving a coronary artery bypass graft (CABG) (65). Goren et al. demonstrated that the circulating level of miR-150 is reduced in patients with AF and systolic heart failure (66).

A means of distinguishing embolic from thrombotic stroke is provided by identification of miRNAs. As we know, patients with AF have a higher probability of stroke, but whether the stroke was caused by AF or atherosclerosis affects clinical decisions, and distinguishing between the two remains a challenge (67). Chen et al. (68) identified miR-15a-5p, miR-17-5p, miR-19b-3p, and miR-20a-5p as biomarkers useful for making this distinction.

#### MicroRNAs in the Mechanism of Atrial Fibrillation

The miR-21-3p in exosomes has been shown to be related to the mechanism of AF (60). And many other miRNAs have been reported to be associated with AF. As mentioned above, AF-related myocardial remodeling can be divided into structural remodeling and electrical remodeling. The earliest research connecting miRNA to myocardial fibrosis can be traced back to van Rooij et al. (69) and Thum et al. (70) in 2008. van Rooij et al. (69) demonstrated that up-regulated miR-29 in fibroblasts can reduce expression of collagen, fibrin, and elastin, thereby reducing the level of fibrosis; Thum et al. (70) found that the level of miR-21 in fibroblasts was selectively increased in heart failure, and miR-21 enhanced ERK-MAP kinase activity by inhibiting (Spry1), thereby enhancing myocardial fibrosis. In the following decade, more and more studies focused on atrial fibrosis and miRNA-21 (71–73). In addition, the pro-fibrotic response of canine atria to nicotine was dependent on down-regulation of miR-133 and miR-590 (74); miR-132 can target connective tissue growth factor in cardiac fibroblasts to regulate fibrosis (75).

In addition to structural remodeling, miRNAs have also been shown to be related to electrical remodeling. Zhao et al. (76) found that miR-29 not only participated in myocardial fibrosis, but was also related to decreased I_ca, L_ density by reducing expression of Ca^2+^ channel subunits. Binas et al. also found that miR-221 and miR-222 participate in cardiac electrical remodeling by regulating expression of L-type Ca^2+^ channel subunits and potassium channel subunits (77).

#### MicroRNAs in the Treatment of Atrial Fibrillation

As mentioned above, miR-320d in exosomes has been shown to decrease apoptosis and increase cell viability in AF, suggesting it could be used for treatment (62).

Some studies have experimented with using miRNA to directly treat AF in animal models. MiR-1 was shown to exacerbate arrhythmia when upregulated in rat hearts, which could be relieved by an antisense inhibitor. Further research revealed that associated mechanisms involve down-regulation of the K^+^ channel subunit Kir2.1 and connexin 43 (78). Other studies have also shown that miRNA can be used to prevent and treat AF in rat, mouse, and canine models (79–81). However, current research remains limited to animal experiments. Safety issues such as off-target effects must be resolved before conducting human trials (82).

### Circular RNAs

Although no studies have yet shown that circRNA in exosomes contributes to the mechanism or is useful for the diagnosis and treatment of AF, many have shown circRNA may have such potential. We expect to see more research on circRNA in exosomes in the future.

#### Circular RNAs in the Diagnosis of Atrial Fibrillation

Shangguan et al. first reported the expression profile of circRNAs in atrial tissues of dogs with AF using high-throughput sequencing. Differentially expressed circRNAs were identified and annotated as being involved in “cytoskeleton structure composition and ion channel activity” (83), laying the foundation for research in this field.

CircRNAs are likely to become more stable and reliable biomarkers for the diagnosis of AF since in contrast to linear RNAs with a 5′ cap and 3′ tail at either end, circRNAs are characterized by a covalent closed loop structure, which may be more stable and conserved (84). In an analysis of human peripheral blood, Ruan et al. (85) identified 5 up-regulated and 11 down-regulated circRNAs and described a molecular regulatory network. Subsequent studies revealed the expression profile and target genes of circRNAs in atrial myocytes of patients with AF. By use of competing endogenous RNA (ceRNA) network analysis, Jiang et al. (86) showed that the circRNAs hsa_circ_0000075 and hsa_circ_0082096 may be involved in the pathogenesis of AF *via* the transforming growth factor (TGF)-beta signaling pathway. Hu et al. (87) identified five circRNAs with significantly large differences (ch9:15474007-15490122, chr16:75445723-75448593, hsa_circ_0007256, chr12:56563313-56563992, and hsa_circ_0003533) and speculated that they are related to inflammation associated with AF. Zhang et al. (88) first reported differences in the expression profiles of circRNAs in the left and right atrial appendages of patients with AF. Liu et al. (89) discovered two key miRNA+circRNA regulatory pathways that may be associated with the mechanisms of AF: hsa-circRNA-100053-hsa-miR-455-5p-TRPV1 and hsa-circRNA-005843-hsa-miR-188-5p-SPON. Wu et al. (90) constructed lncRNA-miRNA-mRNA and circRNA-miRNA-mRNA networks, which enriched the AF molecular network.

The expression profiles of patients with AF and other diseases have also been extensively studied. Zhang et al. (73) showed that plasma Hsa_circRNA_025016 can be used to predict the occurrence of AF after off-pump CABG (91); Hu et al. (92) discussed the expression characteristics of circRNA in patients with rheumatic heart disease and AF. Zhu et al. (93) reported the expression profile of circRNAs related to valvular heart disease and persistent AF.

#### Circular RNAs in the Mechanism of Atrial Fibrillation

It has been reported that circRNAs are involved in the mechanism of AF progression. Gao et al. suggested that circRNAs play an important role in the progression of AF, and identified hsa_circ_0004104 as promoting cardiac fibrosis by targeting the MAPK and TGF-β pathways, and as such is a potential regulator and biomarker of persistent AF (94). Costa et al. demonstrated that an important feature of the progression of paroxysmal to permanent AF is an increase in the expression of circRNAs that can adsorb specific miRNAs, which reduces expression of these post-transcriptional regulatory factors. The targeted miRNA molecules included hsa-miR-181d-5p, hsa- miR-3180-3p, hsa-miR-6868-3p, and hsa-miR-2277-5p (95).

### Long Non-coding RNAs

Among the lncRNAs in exosomes, only the lncRNA NRON has been reported to be involved in AF (61), but many lncRNAs have been extensively studied, and these lncRNAs may become a focus of future research on exosomes. Babapoor-Farrokhran et al. reviewed the role of lncRNAs in AF described in articles published prior to 2020 (96). They concluded that lncRNAs play a variety of roles in the development of AF, including up-regulating or down-regulating atrial structural remodeling, down-regulating electrical remodeling, up-regulating the renin angiotensin system, and reducing abnormalities in calcium handling. However, research in this area is very active and many new studies have emerged since publication of their review.

#### Long Non-coding RNAs in the Diagnosis of Atrial Fibrillation

Studies have shown that lncRNAs can also be used as a biomarker of AF for diagnostic and prognostic evaluation. Shi et al. demonstrated that expression of lncRNA GAS5 was down-regulated in AF patients, which occurred before enlargement of the left atrium, indicating that it can be used as an early biomarker of AF. In addition, they also showed that patients with reduced expression of GAS5 have a higher probability of AF recurring after radio-frequency catheter ablation (97). To be able to predict the risk of stroke in AF patients, Zeng et al. monitored AF patients and compared the levels of the lncRNA ANRIL (antisense non-coding RNA in the INK4 locus) in the stroke group and a non-stroke group. The results showed that higher lncRNA ANRIL levels often correlate with a greater risk of stroke (98).

#### Long Non-coding RNAs in the Mechanism of Atrial Fibrillation

lncRNAs participate in cardiac structural remodeling and electrical remodeling. It has been shown that lncRNA HOTAIR, NRON, LICPAR and MIAT can participate in structural remodeling (61, 99–101). For example, consider the lncRNA HOTAIR, where previous studies demonstrated that miR-613 can target GJA1 to regulate the expression of Cx43, and HOTAIR can sponge miR-613. Consequently Dai et al. speculated that HOTAIR could act as a ceRNA to regulate Cx43 expression in AF by sponging miR-613. They collected atrial tissue from patients with valvular heart disease and cultured HL-1, a mouse atrial cell line, and using RT-PCR, western blot and luciferase activity assays, found that the suppressive effect of miR-613 on Cx43 expression was attenuated by HOTAIR. Their study identified a novel HOTAIR/miR-613/Cx43 axis in the regulation of AF, which has the potential to be a therapeutic target (99).

In addition, lncRNA can participate in the electrical remodeling. Du et al. found that expression of the lncRNA TCONS-00106987 was significantly higher in AF rabbit models compared with non-AF rabbit models. Then they infected the rabbits with lentiviruses that mediated over-expression of TCONS-00106987 and found that they were more prone to AF. Further research confirmed that TCONS-00106987 promoted electrical remodeling *via* sponging miR-26 to increase expression of the gene KCNJ2, thereby increasing inward-rectifier K^+^ current (102). The lncRNAs and their functional mechanisms summarized in the Table 2.

**Table 2:** The lncRNAs and their functional mechanisms on AF.

**Model**	**LncRNA**	**Effect of AF**	**Targets and mechanisms**	**References**
Human/Mice	HOTAIR	Alleviates	Regulates Cx43 remolding by sponging miR-613.	(99)
Mice	NRON	Alleviates	Promotes M2 macrophage polarization and alleviates atrial fibrosis through suppressing exosomal miR-23a.	(61)
Human	LICPAR	Aggravate	Modulates TGF-β/Smad pathway.	(100)
Human/Rat	MIAT	Aggravate	MIAT downregulation alleviates AF and AF-induced myocardial fibrosis through targeting miR-133a-3p.	(101)
Rabbit	TCONS-00106987	Aggravate	Promotes atrial electrical remodeling by sponging miR-26 to regulate KCNJ2.	(102)

## Exosome-Mimetic Nanovesicles and Engineered Nanoparticles

However, there are still obstacles to the clinical application of exosomes in AF. First, isolation of exosomes with high yield, reproductivity and purity is challenging (103). Second, we also lack efficient methods of loading drugs into the exosomes. Third, although numerous reports have shown that exosomes can be used as biomarkers for AF, the results from different studies are often inconsistent. One explanation may lie in the different conditions of patients in the different studies, and another may be methodological differences in exosome purification (104). The potential solution may be to more precisely define experimental conditions and to combine multiple components as biomarkers in exosomes to manage exosome heterogeneity among patients. To successfully use exosomes for drug delivery, the problem of their low yield must be solved. For this reason, generation of exosome-mimetic vesicles or engineered nanoparticles with a substantially greater yield has attracted recent attention.

### Exosome-Mimetic Nanovesicles

Jang et al. (105) developed a method to load chemotherapeutics into bioinspired exosome-mimetic nanovesicles for delivery to tumor tissue. Monocytes or macrophages were forced to pass sequentially through filters of diminishing pore size, which caused the cells to break up and release chemotherapeutics-loaded nanovesicles. Surprisingly, these nanovesicles possessed characteristics of exosomes, such as targeting ability, and they were also shown to have anti-tumor properties. More importantly, this method increased the yield by 100-fold compared with exosomes, suggesting that these bioengineered nanovesicles can effectively deliver chemotherapeutics to treat malignant tumors. Similarly, Yoon et al. (106) generated nanovesicles by slicing living cell membranes with microfabricated 500 nm-thick silicon nitride blades, and successfully delivered exogenous substances to recipient cells. These results suggest that exosome-mimetic nanovesicles can deliver drugs on a large scale.

### Engineered Nanoparticles

Recently, engineered nanoparticles, such as fluorescent nanodiamonds, magnetic iron oxide nanoparticles, and silica nanoparticles, have attracted enormous attention due to their great potential for applications in medicine (107–109). Compared with natural vesicles, these artificially synthesized particles are more stable and easier to synthesize *in vitro*. In addition, nanoparticles have many useful characteristics, including optical and electromagnetic properties, lacking in natural vesicles (110). Researchers can also adjust the properties of nanoparticles by changing their size (110). For example, particles smaller than 12 nm can pass through the blood-brain barrier, and they can be endocytosed by cells when <30 nm. Shape and surface charge can alter particle affinity for specific organs and tissues (111). As a result, there is much research interest in the application of engineered nanoparticles to the diagnosis and treatment of disease. For example, multifunctional magnetic iron oxide nanoparticles are applied to magnetic hyperthermia treatment and photothermal therapy of tumors (112). Although there are few studies on the application of nanoparticles to AF, we can envision nanoparticles being used as contrast agents for imaging lesions in the cardiovascular system and as carriers for drug delivery. However, since nanoparticles are artificially synthesized, it will be necessary to carefully explore their toxicity and other potential adverse effects before being used in the clinic (113).

## Conclusion

Exosomes provide a new perspective on AF. With greater stability, higher enrichment, and most importantly, greater targeting ability than non-specific drugs, exosomes are potentially useful diagnostic biomarkers and delivery vehicles for treating AF. Nevertheless, in order to make them useful in clinical practice, several limitations should be overcome. First, there are few existing studies of exosomes in the context of AF, but there are many studies of ncRNAs. Future research on exosomes should focus on ncRNA molecules that have been associated with AF. Second, we lack effective methods for extracting and purifying exosomes, resulting in low exosome yield. To solve this problem, we need, on the one hand, to optimize existing extraction technology, while on the other hand, to take advantage of artificial drug delivery systems such as exosome-mimetic nanovesicles and engineered nanoparticles. In summary, if we carry out detailed research on exosomes, conduct larger clinical trials, and increase yields, exosome-based diagnosis and therapy could greatly improve current management of patients with AF.

## Author Contributions

SH wrote the manuscript. SH, YD, and JX carried out the data collection and/or assembly of data and data analysis. JL and LL carried out data analysis and interpretation, and contributed to manuscript revisions. CF was responsible for conception and design of the study and contributed to manuscript revisions. All authors read and approved the final manuscript.

## Conflict of Interest

The authors declare that the research was conducted in the absence of any commercial or financial relationships that could be construed as a potential conflict of interest.

## Publisher's Note

All claims expressed in this article are solely those of the authors and do not necessarily represent those of their affiliated organizations, or those of the publisher, the editors and the reviewers. Any product that may be evaluated in this article, or claim that may be made by its manufacturer, is not guaranteed or endorsed by the publisher.
